# Propagermanium as a Novel Therapeutic Approach for the Treatment of Endothelial Dysfunction in Type 2 Diabetes

**DOI:** 10.3390/ijms25158328

**Published:** 2024-07-30

**Authors:** Lara Azul, Adriana Leandro, Raquel Seiça, Cristina M. Sena

**Affiliations:** Institute of Physiology, iCBR, Faculty of Medicine, University of Coimbra, Subunit 1, Polo 3, Azinhaga de Santa Comba, Celas, 3000-548 Coimbra, Portugal

**Keywords:** propagermanium, type 2 diabetes, endothelial dysfunction, inflammation, insulin resistance

## Abstract

Propagermanium (PG) has immune modulating activity and anti-inflammatory properties. This work aimed to study the therapeutic efficacy of PG on endothelial and perivascular dysfunction associated with type 2 diabetes. Non-obese type 2 diabetic Goto-Kakizaki (GK) rats were divided into four groups: (1) the control group; (2) the group treated with 50 mg/kg PG; (3) the group fed a high-fat diet (GKHFD); and (4) the group of GKHFD treated with 50 mg/kg PG. PG was given orally for 3 months. Several in vivo parameters and endothelial function were studied in aortas with perivascular adipose tissue PVAT (+) or without PVAT (−). We also determined the vascular inflammation and levels of CD36 in PVAT. In diabetic GK rats, PG did not affect the lipid profile or the results of the intraperitoneal glucose tolerance test. Instead, it improved the fasting glucose levels (18%, *p* < 0.01), insulin resistance (32%, *p* < 0.05), endothelial function (33 and 25% in aortas mounted with (+) or without PVAT (−), *p* < 0.05), and restored the anticontractile effect of the perivascular adipose tissue by reducing its inflammation (56%, *p* < 0.05) and oxidative stress profile (55%, *p* < 0.05). Due to its anti-inflammatory characteristics, PG likely improved endothelial dysfunction and restored the perivascular adipose tissue’s anticontractile properties.

## 1. Introduction

Type 2 diabetes (T2D) is associated with an increased risk of cardiovascular disorders and major vascular complications [1,2,3]. One of the earliest pathophysiological events in the development of atherosclerosis is endothelial dysfunction, which is characterized by abnormal vascular reactivity brought on by a decrease in nitric oxide (NO) bioavailability along with a change in the characteristics of the endothelium toward reduced vasodilation, a thrombogenic and proinflammatory state [4]. A series of events in the arterial wall are brought about by diabetes, including increased oxidative stress, endothelial dysfunction, low-grade inflammation, and the dysfunction of the perivascular adipose tissue (PVAT) [4,5,6].

An important factor in the development and progression of atherosclerosis is vascular inflammation. In vascular inflammation and remodeling, chemokine C-C motif chemokine 2 (CCL2, also called monocyte chemoattractant protein 1) and its receptor, C-C chemokine receptor type 2 (CCR2), form a significant and important signaling pathway. The recruitment of macrophages and monocytes to inflammatory sites in both adipose tissue and the vasculature is facilitated by the CCL2/CCR2 pathway [7,8]. Insulin resistance and type 2 diabetes are linked to the recruitment of macrophages in adipose tissue depots [9].

This recruitment of macrophages in adipose tissue depots is associated with the onset of insulin resistance and type 2 diabetes [9]. In Japan, hepatitis B is treated with propagermanium (PG), a water-soluble organogermanium compound that is a polymer of 3-oxygermyl propionic acid [10]. Due to its interaction with CCR2-associated glycosylphosphatidylinositol-anchored proteins, PG displays immunomodulatory effects. PG stifles monocyte–macrophage chemotaxis by blocking the CCL2/CCR2 signaling pathway, while maintaining the receptor–ligand interaction [11].

PG exhibits therapeutic effects in a range of inflammatory diseases, including obesity caused by a high-fat diet [7,12,13], fibrosis [14,15,16], and atherosclerosis [17,18,19], and prevents insulin resistance and steatosis in wild-type mice [20] and db/db mice [12]. Furthermore, PG administration has demonstrated efficacy in eliminating inflammatory conditions, such as experimental atherosclerosis, that are primarily mediated by inflammatory monocytes and macrophages [17,18]. Previous research [18] demonstrated that PG significantly suppressed the formation of coronary arteriosclerotic lesions in vivo by the macrophage-mediated chemotaxis of porcine monocytes induced by CCL2 at clinical concentrations. These findings raise the possibility of PG’s application in the treatment of arteriosclerotic vascular diseases.

PG conveys therapeutic effects in a range of inflammatory conditions. It is still unknown how PG therapy affects PVAT characteristics and vascular dysfunction in T2D. Here, we sought to examine the effects of PG on vascular inflammation in the aorta of eight-month-old Goto-Kakizaki (GK) rats as well as endothelial dysfunction and the PVAT phenotype. We assessed the function of the endothelium and described the alterations noted in the inflammatory and vasoconstriction profiles of PVAT. To the best of our knowledge, we are the first to assess how PG administration affects the function of the aortic endothelium and the periaortic adipose tissue in T2D.

## 2. Results

### 2.1. Characteristics of the Animal Models

Throughout this study, there was no variation in the weekly amount of food consumed between the groups. The body weight gain in the GK rats fed a high-fat diet (GKHFD) was significantly increased (5%). In the GK and GKHFD groups, PG treatment had no visible effect on body weight (Table 1). The levels of the total cholesterol and triglycerides were considerably higher in the GKHFD rats. In the GKPG group, the lipid profile did not change. The triglyceride levels considerably dropped (18%) and the total cholesterol levels rose by 22% in GKHFDPG rats when compared to the GKHFD group (Table 1).

The PG treatment significantly decreased the blood urea nitrogen levels in the GK group. The uric acid levels were similar between the experimental groups (Table S1). The alkaline phosphatase (ALP) levels were significantly increased in the diabetic GKHFD group. The serum aspartate aminotransferase (AST) and alanine aminotransferase (ALT) levels were not significantly different between the GK and GKHFD groups. The serum ALT and ALP levels were significantly reduced in the GKPG and GKHFDPG groups when compared with the respective controls (Table S1). All these data indicate that PG does not exhibit any hepatic or renal toxicity.

**Figure 1:**
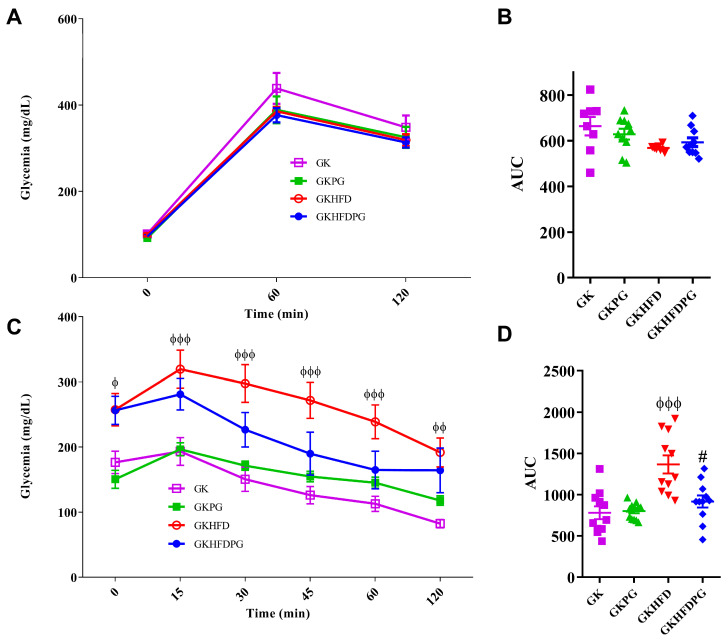
Influence of propagermanium on blood glucose levels during an intraperitoneal glucose tolerance test (IPGTT; (**A**)), the glucose area under the curve (AUC; (**B**)), insulin tolerance test (ITT; (**C**)), the insulin area under the curve (AUC; (**D**)) in 8-month-old diabetic Goto-Kakizaki (GK) control rats and GK rats fed with high-fat diet (GKHFD). GK and GKHFD rats were treated with propagermanium (50 mg/kg/day, orally) for 3 months (GKPG and GKHFDPG, respectively). The AUC of ITT and IPGTT were evaluated to determine the grade of insulin resistance and glucose tolerance impairment, respectively. Data are expressed as mean ± SE (n = 12). ^ϕ^ *p* < 0.05, ^ϕϕ^ *p* < 0.01, ^ϕϕϕ^ *p* < 0.001 vs. GK group; ^#^ *p* < 0.05 vs. GKHFD group.

The diabetic GK and GKHFD groups exhibited glucose intolerance in an intraperitoneal glucose tolerance test (IPGTT), as observed in Figure 1A. The glucose area under the curve (AUC) was comparable across all groups and did not vary with PG (Figure 1B).

Insulin resistance was evaluated in diabetic GK rats using the insulin tolerance test (ITT); this was found to be significantly deteriorated in the GKHFD group, as indicated by the large increase in the AUC (Figure 1C,D). In the GKHFDPG group, the ITT and AUC were significantly lower (Figure 1C,D).

### 2.2. Vascular Relaxation in the Rat Aorta Dependent on NO

**Figure 2:**
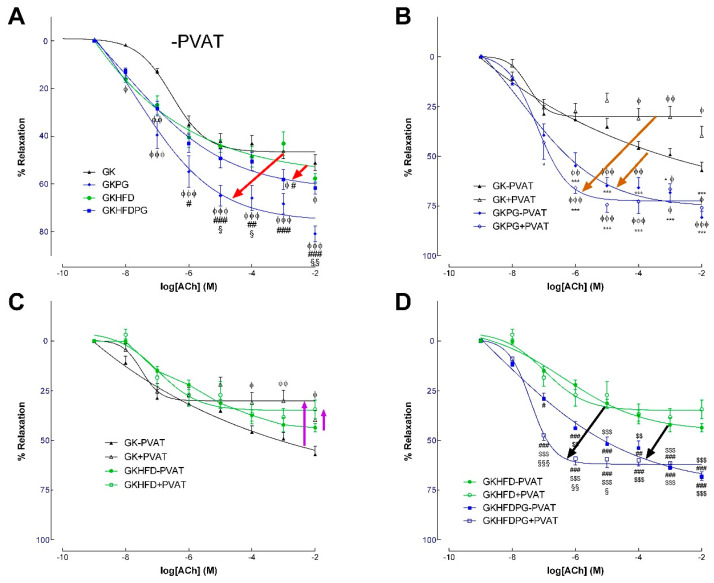
The impact of propagermanium and perivascular adipose tissue (PVAT) on vasodilatory responses to acetylcholine (ACh) in the aortas of GK control rats (GK) and GK fed with high-fat diet (GKHFD). Endothelium-dependent vasodilation in aortic rings in the absence (−PVAT, (**A**)) or presence of PVAT (+PVAT; (**C**)) was assessed in GK and GKHFD with or without propagermanium treatment (**A**–**D**). Panels (**B**,**D**) illustrate the impact of PVAT on GK control and GKHFD rats, respectively. In GK and GKHFD rats, propagermanium partially restored the impaired ACh-induced relaxation (the propagermanium effects are indicated by the arrows). In the GK+PVAT aorta, this effect is more pronounced (arrows in panel (**B**)). Data are expressed as mean ± SE (n = 12). ^ϕ^ *p* < 0.05, ^ϕϕ^ *p* < 0.01, ^ϕϕϕ^ *p* <0.001 vs. GK−PVAT group; * *p* < 0.05, *** *p* < 0.001 vs. GK+PVAT group; ^#^ *p* < 0.05, ^##^ *p* < 0.01, ^###^ *p* < 0.001 vs. GKHFD−PVAT group; ^§^ *p* < 0.05, ^§§^ *p* < 0.01, ^§§§^ *p* < 0.001 vs. GKPG−PVAT group. ^$$^ *p* < 0.01, ^$$$^ *p* < 0.001 vs. GKHFD+PVAT.

**Figure 3:**
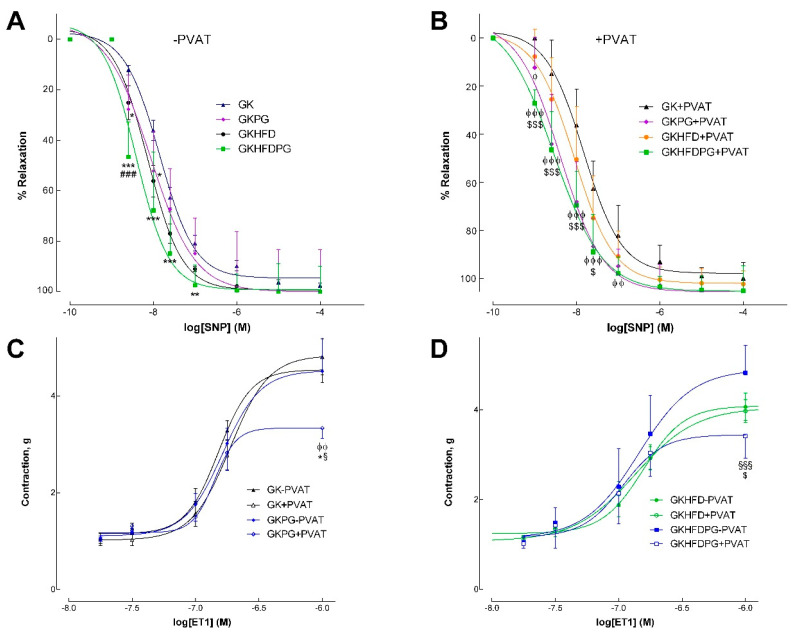
The effects of propagermanium and perivascular adipose tissue (PVAT) on vasodilatory responses to sodium nitroprusside (SNP) and contraction responses to endothelin–1 (ET1) in the aortas of GK control rats (GK) and GK fed with high-fat diet (GKHFD). Endothelium-independent vasodilation in aortic rings in the absence (−PVAT) or presence of PVAT (+PVAT) was assessed in GK and GKHFD with or without propagermanium treatment (**A**,**B**). The effect of treatment with propagermanium in the vascular contraction to ET1 in GK and GKHFD rats, in the absence (−PVAT) or presence of PVAT (+PVAT), is displayed in panels (**C**,**D**). In diabetic GK (panel (**C**)) and GKHFD rats (panel (**D**)), propagermanium partially restored the anticontractile PVAT phenotype characteristic of normal rats. Data are expressed as mean ± SE (n = 12). ^ϕ^ *p* < 0.05, ^ϕϕ^ *p* < 0.01, ^ϕϕϕ^ *p* < 0.001 vs. GK−PVAT group; * *p* < 0.05, ** *p* < 0.01, *** *p* < 0.001 vs. GK+PVAT group; ^###^ *p* < 0.001 vs. GKHFD−PVAT group; ^§^ *p* < 0.05, ^§§§^ *p* < 0.001 vs. GKPG−PVAT group. ^$^ *p* < 0.05, ^$$$^ *p* < 0.001 vs. GKHFD+PVAT.

Endothelium-dependent and -independent relaxations of phenylephrine-precontracted aorta arterial rings were measured with (+) or without (−) periaortic adipose tissue (PVAT of aorta) mounted on a four-channel wire myograph. In GK−PVAT and GK+PVAT, the maximal endothelium-mediated relaxation of the phenylephrine-precontracted rings decreased by 47% and 66%, respectively, in response to ACh (Figure 2A,B). Although endothelium-mediated relaxation declined in GKHFD−PVAT by 53% and GKHFD+PVAT by 65%, respectively, Table 2 shows that a high-fat diet did not significantly alter this impairment. In GK and GKHFD rats (Figure 2A), PG treatment markedly enhanced endothelium-dependent vascular relaxation in arteries with (+) or without (−) PVAT (Figure 2B,D). When PVAT surrounds the arteries (GKPG+PVAT and GKHFDPG+PVAT; Figure 2B,D), the effect is more pronounced. In fact, a notable rise in ACh sensitivity without appreciable alterations to maximal relaxation was noted when ACh-induced relaxation was detected in aortic segments from GKHFDPG+PVAT (Figure 2D, Table 2, *p* < 0.001). When compared to GK, GK+PVAT, GKHFD, and GKHFD+PVAT, vascular sensitivity to ACh was markedly enhanced in GK rats with PG (GKPG; GKHFDPG; Figure 2A, Table 2). The concentration–effect curves to SNP showed no group differences in maximal relaxation (Figure 3A,B; Table 2). The aorta of GK rats given PG showed a significant improvement in vascular sensitivity to SNP (Figure 3A,B, Table 2). In GK rats, relaxation to ACh was nearly complete by preincubating the aorta with indomethacin and L-NAME. Table 2 provides a detailed summary of the EC50 values and maximal relaxations.

### 2.3. Response of the Arteries to Endothelin–1

A four-channel wire myograph was used to mount aortas with (+) or without (−) periaortic adipose tissue to perform cumulative concentration–response curves to endothelin–1 (ET1). The aortas of diabetic GK rats that have been mounted with PVAT do not exhibit an anticontractile profile that would alter ET1’s maximal contraction (Figure 3C,D). The ET1 contraction responses in the aorta of diabetic rats given a high-fat diet but not PVAT or PG treatment were not significantly altered (Figure 3C,D). Aortic rings with PVAT dramatically decreased contraction to endothelin–1 in GKPG (31%) and GKHFDPG (27%) rats, demonstrating the potent anticontractile action of thoracic periaortic PVAT (Figure 3C,D). Table 2 displays the maximal contractions and EC50 values. The anticontractile effect of thoracic periaortics in animals is demonstrated by the fact that there are no changes in the EC50 values in the dose–response curves to ET1, and maximal contractions in the GKPG+PVAT and GKHFDPG+PVAT arteries are significantly reduced (*p* < 0.01; Figure 3C,D).

### 2.4. Vascular Wall Inflammation

**Figure 4:**
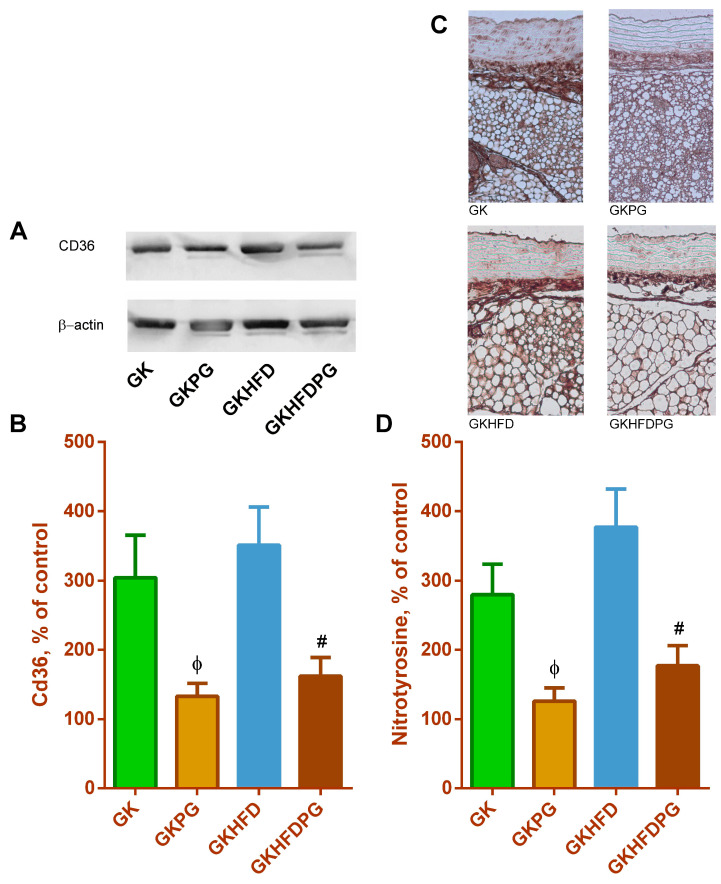
The effects of propagermanium on inflammation of perivascular adipose tissue (PVAT) in thoracic aorta from diabetic Goto-kakizaki control (GK) and GK fed with high-fat diet (GKHFD) rats. Inflammatory tissue CD36 (**A**,**B**) levels and immunohistochemical staining for nitrotyrosine (**C**,**D**) were determined in PVAT of thoracic aortas of GK and GKHFD rats treated with propagermanium for 3 months. Sample blots representative of 5 independent experiments with identical results. Data are expressed as mean ± SE (n = 12). ^ϕ^ *p* < 0.05 vs. GK group; ^#^ *p* < 0.05 vs. GKHFD group.

The periaortic adipose tissue of diabetic GK rats exhibits a significant increase in the biomarkers of inflammation, CD36 and CCL2. Furthermore, a rise in the nitrotyrosine levels in diabetic rats’ PVAT was also documented, supporting the rise in the inflammatory and oxidative stress profile in the rats’ periaortic adipose tissue. Here, we confirm that periaortic adipose tissue has a pro-inflammatory profile (shown by an increase in CD36 levels, Figure 4A). We also show that PG treatment significantly reduced periaortic CD36 levels, along with a decrease in PVAT nitrotyrosine levels (Figure 4B), which was observed in both diabetic GK groups. This reduced the pro-inflammatory and pro-oxidant profile of PVAT. The immunoreactive nitrotyrosine levels in the aortas of diabetic vasculature were found to be twice as high (Figure 4). The vascular nitrotyrosine levels were substantially lowered by PG treatment (Figure 4B).

## 3. Discussion

According to earlier research, blocking the CCL2/CCR2 pathway could be a secure and useful treatment option for vascular disease [20,21]. The effects of PG treatment on periaortic adipose tissue in type 2 diabetes and its vascular dysfunction in vivo have not been studied. In this work, we demonstrate that PG ameliorates the endothelial function in an animal model of type 2 diabetes by significantly reducing inflammation in PVAT and restoring its anticontractile phenotype. For the first time, we assessed the effects of PG treatment in vivo on the periaortic PVAT and vascular function of diabetic GK rats, which helped to clarify the mechanisms behind this therapeutic strategy.

Research in animal models has demonstrated the efficacy of PG in the treatment of diabetes [7,22], nephropathy [23], and inflammation of the kidney, liver, and brain [13,14,24,25]. Furthermore, prior studies have demonstrated that PG effectively reduced hepatic steatosis in obese [13] and db/db mice [12]. In db/db mice at 18 weeks of age, but not at 9 weeks, PG reduced the hepatic expression of CD36, which uptakes FFA into the liver, and hepatic FFA [12]. These results imply that propagermanium reduces the amount of FFA that enters the liver of db/db mice [12]. In addition, it was previously shown that PG reduces oxLDL levels in vitro and in vivo [26,27]. We observed an increment in the total cholesterol that may be due to an increment in the HDL cholesterol levels.

The preservation of insulin sensitivity and the overall metabolism is strongly influenced by inflammation [7,28]. Previous research has shown that an increase in inflammatory cells in the liver and white adipose tissue occurred concurrently with the development of insulin resistance [29]. Insulin resistance is also influenced by changes in the phenotype of macrophages in addition to variations in the quantity of inflammatory cells. In a mouse model fed a high-fat diet, it was previously demonstrated that PG improved non-alcoholic steatohepatitis, white adipose tissue inflammation, and insulin resistance [13] and earlier interventions were more effective. Accordingly, we demonstrate that PG treatment dramatically lowers insulin resistance in GKHFD rats, emphasizing the critical role that inflammation plays in regulating insulin sensitivity.

The correlation between fatty liver and cardiovascular complications observed in epidemiological studies is mediated, among others, by insulin resistance and dyslipidemia, two established risk factors for atherosclerosis [29]. The potential link between the two disorders is oxidative stress and inflammatory processes [29]. PG effectively ameliorates both insulin resistance and the lipid profile [12,13,22]. Herein, we show that PG treatment reduces both oxidative stress and inflammation in PVAT, therefore being able to tackle both mechanisms crucial to vascular dysfunction in type 2 diabetes. In addition, other tissues and organs are probably involved including the liver, kidneys, and immune system (not evaluated in this study). Altogether, due to its antioxidant and anti-inflammatory properties, PG reduces the insulin resistance and endothelial dysfunction associated with type 2 diabetes. In animal models of atherosclerosis, propagermanium inhibits macrophage infiltration by blocking the CCR2 function [17,18,19]. Furthermore, CCL2, IL-8, CCL5, and CCR2 expression are among the other chemokines whose functions are unaffected by PG [11,30,31]. In T2D patients, low-grade inflammation has been connected to endothelial dysfunction [32,33]. In Apo-E knockout mice, PG inhibits macrophage infiltration, preventing the formation of atherosclerotic lesions, according to research by Yamashita and colleagues [11]. Here, we discovered that PG treatment significantly reduced the relaxation of the aorta brought on by ACh in GK and GKHFD rats, thereby improving endothelial dysfunction in GK rats with diabetes. In arteries without PVAT, PG did not affect SNP-induced, endothelial-independent vasorelaxation. Furthermore, PG significantly recovered the anticontractile effect of PVAT in the diabetic GK aortas in response to the ET1-induced vasoconstrictor.

The vasculature is negatively impacted by the inflammation of perivascular adipose tissue [33,34] due to the presence of macrophages, which express and release various factors, including cytokines. The cytokines may also originate from visceral adipose tissue, which can cause liver inflammation through portal circulation. This can also trigger a chronic and systemic inflammatory response that damages endothelial cells. One molecule that reduces inflammation is PG [11].

By encouraging the recruitment of monocytes and macrophages to inflammatory sites, CCR2 regulates the immune response and is linked to the development of diabetes [35,36,37]. The development of insulin resistance and type 2 diabetes is significantly influenced by macrophage recruitment into adipose tissue via CCR2 [38]. In the current study, PG treatment was able to significantly lower CD36 levels in the PVAT of diabetic GK rats, improving the periaortic adipose tissue phenotype.

Notably, the PG dosage in this study is comparable to that which is clinically administered for treatment (30 mg/d is the clinically recommended dose [10]). Our study shows that PG treatment was beneficial, significantly decreasing the ALT and ALP levels. Furthermore, in GK control rats, PG does not alter the CCR2 expression levels in periaortic adipose tissue (Figure S1), which is consistent with other findings in apoE−/− mice’s macrophages or atherosclerotic lesions. By binding to the CCR2 (via the N-terminal peptides) and a glycosylphosphatidylinositol (GPI)-anchored protein, PG inhibits the CCR2 function [11].

TLK–19705, a CCR2 antagonist, was tested by Okamoto and colleagues [39] for its impact on atherosclerosis in mouse models of diabetic nephropathy. Both in vitro and in vivo, this antagonist prevents the chemotaxis that CCL2 induces. In apoE−/− mice, supplementation with TLK–19705 reduced the area of atherosclerotic lesions. In THP–1 cells, PG produced similar outcomes [11]. By restricting CCL2/CCR2 activity, PG prevented tissue damage in mice by inhibiting monocyte recruitment [17]. These findings imply that both antagonists can be helpful therapeutic agents for inflammatory diseases.

However, unsatisfactory research on the inhibition of the CCL2/CCR2 axis was reported [40,41]. When ApoE−/− mice were treated with INCB–3344, a CCR2 small-molecule antagonist, the size and progression of the atherosclerotic lesions did not decrease. The endogenous murine CCR2 gene was substituted with human CCR2 in an animal model of atherosclerosis fed a Western diet and given angiotensin II infusion [40]. The authors then examined the impact of the CCR2 antagonist GSK1344386B on the development of atherosclerotic plaque. The aortic root lesion area did not decrease, but the authors did note a 30% reduction in the macrophage area in these animals. These investigations imply that there is conflicting evidence regarding these small molecules’ antagonistic effectiveness in cardiovascular disease. Restrictions concerning the therapeutic benefits of CCL2/CCR2 antagonists and antibodies in the management of cardiovascular disorders encompass the assessment of off-target consequences, toxicity [21,42], and the possibility of compensatory activity by alternative receptors. All things considered, our research suggests that PG may find use in the management of vascular diseases associated with diabetes. Therefore, the PG modulation of the CCL2/CCR2 pathway may be a safe and useful therapeutic strategy for the prevention of atherosclerosis.

This study has several limitations. It was conducted exclusively in 8-month-old male diabetic GK rats, which may limit the generalizability of the findings to other age groups, female rats, and different models of diabetes. The further evaluation of the liver, renal, and spleen histology, along with the assessment of the impact of PG on inflammation and oxidative stress in these organs, would provide a clearer understanding of the mechanisms underlying its effects. Moreover, the determination of lipoprotein subfractions would be important for a comprehensive analysis, as it would offer more detailed insights into the lipid metabolism and additional cardiovascular implications in this model.

## 4. Material and Methods

### 4.1. Materials

Phenylephrine (P6126), propagermanium (12758-40-6), acetylcholine (A6625), and N-nitro-L-arginine- methyl ester (L-NAME; N5751) were obtained from SIGMA (St. Louis, MO, USA). Antibody against nitrotyrosine Upstate^®®^ (06-284) was obtained from Merck (Darmstadt, Germany). Antibodies against β-actin (ab8226) and cluster of differentiation 36 (CD36) (ab252923) were obtained from Abcam plc (Cambridge, UK). Ketamine and chlorpromazine chloride were obtained from Parke-Davis, Ann Arbor, MI, USA, and Lab. Vitória, Portugal, respectively. All other chemicals and reagents used in this study were of high grade.

### 4.2. Animals

Portuguese Law on Experimentation with Laboratory Animals, which was derived from Directive 2010/63/EU, was followed when handling animals and conducting experiments. The Faculty of Medicine in Coimbra, Portugal, provided adult male GK rats for the experiments, and the local Animal Welfare Committee (ORBEA No. 25/2015) approved them in compliance with ARRIVE guidelines.

Male GK diabetic rats (mean body weight 294 g) were separated into four groups for the experiments. (1) Control group (GK); (2) Group administered with PG (50 mg/kg/day, orally) for 3 months (GKPG); (3) Group fed with high-fat diet (20% cocoa butter, 1.25% cholesterol; Safe E8220 version 151, Augy, France) for 5 months (GKHFD); (4) GKHFD group with PG (50 mg/kg/day, orally) for 3 months (GKHFDPG). The acclimation time before starting the protocol was 1 week. The rats were fed a standard commercial pellet diet (Diet AO3; Panlab), had unrestricted access to water, and had 12 h cycles of light and darkness.

Rats were anesthetized when they were 8 months old and blood, aortas, and PVAT were taken (see below).

### 4.3. Glucose and Insulin Tolerance Tests and Lipid Profile

Following an overnight fast, GK rats were rendered unconscious using a combination of ketamine and chlorpromazine (75 mg/kg of ketamine chloride and 2.65 mg/kg of chlorpromazine chloride, intraperitoneally), and they were then killed by cervical dislocation. Tests for insulin and glucose tolerance were conducted as before [5]. Briefly, rats were fasted for the entire night before receiving an injection of glucose (i.p.; 1.75 g kg^−1^) in PBS for glucose tolerance testing. Using a glucometer (Glucometer-Elite-Bayer, Portugal S.A.) and reactive test strips that were compatible, blood glucose was measured by taking a sample from the tail vein at 0, 60, and 120 min after injection. Rats were fasted for 6h before insulin (0.25 U mL^−1^ kg^−1^) was injected for insulin tolerance testing, and glucose was measured as previously described [5]. Using commercially available kits (Sigma-Aldrich Co (St. Louis, MO, USA), lipids (total cholesterol and triglycerides) were measured [5].

### 4.4. Isometric Tension Studies

Aortas were quickly removed and cleaned in a modified Krebs–Henseleit buffer (pH 7.4) at 37 °C (concentrations in mM: NaCl 119; KCl 4.7; MgSO_4_ 1.2; CaCl_2_ 1.6; NaHCO_3_ 25; KH_2_PO_4_ 1.2; glucose 11.0). Then, the segments were separated into two (4 mm width) with or without PVAT and mounted in a wire myograph (DMT myograph system, DK) using an oxygenated (95% O_2_, 5% CO_2_) solution. After stabilizing for 45 min with a resting tension of 4.7 mN, 0.3 µM phenylephrine was used to pre-constrict all the vessels. As previously reported [5], concentration-dependent relaxation to sodium nitroprusside (SNP, 10^−9^ to 10^−4^ M), acetylcholine (ACh, 10^−9^ to 10^−2^ M), or endothelin–1 was obtained.

### 4.5. Analysis Using Western Blot

Rinsing and chilling the tissues in a buffer containing the following in mM: Tris–HCl 50, NaCl 150, EGTA 0.1, EDTA 1, SDS 0.1%, NP-40, 0.1%, and deoxycholate 0.5%, 1 mM phenylmethylsulfonyl fluoride, 10 μg/mL aprotinin, and 10 μg/mL pepstatin. Leupeptin (10 μg/mL) is included. The tissues were homogenized quickly and then centrifuged at 14,000× *g* for 20 min at 4 °C. The concentration of total protein was measured after collecting the supernatants. Then, 20 μg of protein samples were electroblotted onto polyvinylidene difluoride membrane after being separated and loaded onto a sodium dodecyl sulfate polyacrylamide gel electrophoresis (SDS-PAGE). Membranes were blocked, as previously mentioned [5], and then incubated with primary antibodies and alkaline phosphatase secondary antibodies for an entire night. As in the past, immunoblots were later developed [5]. Pierce protein assay kit (23225; Thermo Fisher scientific, Waltham, MA, USA) was used to quantify protein content. Western blots were quantified as previously [5].

### 4.6. Histology

After administering anesthesia, rats received a two-minute transcardiac saline perfusion. For fifteen minutes, the arterial system was perfusion-fixed with 4% paraformaldehyde that had been buffered with phosphate (pH 7.4). After being dissected, the aorta was fixed for an additional overnight in 4% paraformaldehyde in preparation for immunohistochemical examination. For histological analysis, tissue sections (4 μm) were immunostained for nitrotyrosine and stained with hematoxylin and eosin (H&E).

The histological assessment was conducted by an independent investigator who was blinded to the treatment conditions using ImageJ software v.1.45.

### 4.7. Statistical Analysis

GraphPad Prism PC Software (version 3.0) was used to analyze data [expressed as mean ± SE (n = 12 rats per group)]. Significant differences were assessed using one-way ANOVA followed by Bonferroni post hoc test for individual comparisons. *p* < 0.05 was considered significant. Dose–response curves were fitted by nonlinear regression with simplex algorithm. Dose–response curves were appraised by two-way ANOVA for repeated measures followed by Bonferroni post hoc test for individual comparisons.

## 5. Conclusions

Probably because of its anti-inflammatory qualities, PG restored the vasodilating phenotype of perivascular adipose tissue and improved endothelial dysfunction. Perivascular adipose tissue is a possible therapeutic target for the vascular dysfunction associated with type 2 diabetes because it plays a role in the regulation of endothelial function and vascular contractility.

## Figures and Tables

**Table 1:** Body weights, fasting glucose levels, triglycerides, and total cholesterol levels of eight-month-old diabetic Goto-Kakizaki (GK) control rats, GK rats fed a high-fat diet (GKHFD), and GK rats treated with propagermanium (GKPG, GKHFDPG).

	GK	GKPG	GKHFD	GKHFDPG
Body weight (g)	383 ± 4.5	386.1 ± 6.5	401.9 ± 3.7 ^ϕ^	417.6 ± 9.4 ^ϕϕ^
Fasting glucose (mg/dL)	96.6 ± 3.3	90 ± 3.2	99 ± 3.1	81.5 ± 3.5 ^ϕϕ §§§ ##^
Triglycerides (mg/dL)	143.1 ± 4.3	143.2 ± 5.4	168.9 ± 10.2 ^ϕϕ^	141.0 ± 9.2 ^#^
Total cholesterol (mg/dL)	157.7 ± 1.3	158.8 ± 1.4	168.7 ± 4.5 ^ϕ^	203.6 ± 11.2 ^ϕϕ ##^

The data are presented as mean ± SE (n = 12 animals in each group). ^ϕ^ *p* < 0.05, ^ϕϕ^ *p* < 0.01, vs. GK rats; ^#^ *p* < 0.05, ^##^ *p* < 0.01 vs. GKHFD; ^§§§^ vs. GKPG.

**Table 2:** In isolated aorta arteries of eight-month-old diabetic Goto-Kakizaki (GK) control rats, GK rats fed a high-fat diet (GKHFD), and GK rats treated with propagermanium (GKPG, GKHFDPG) with (+PVAT) or without (−PVAT) perivascular adipose tissue, the maximal relaxation responses (%) and −logEC50 were measured. The negative logarithm (−logEC50) of the agonist’s concentration is used to display pEC50 values.

	GK−PVAT	GK+PVAT	GKPG−PVAT	GKPG+ PVAT	GKHFD−PVAT	GKHFD+ PVAT	GKHFDPG−PVAT	GKHFDPG+ PVAT
ACh								
pEC_50_	6.5 ± 0.14	6.2 ± 0.26	7.69 ± 0.8	7.2 ± 0.17	6.35 ± 0.22	6.97 ± 0.23	8.6 ± 1.5	7.41 ± 0.09
Maximal relaxation (%)	52.6 ± 5.1	34.04 ± 6.2 ^ϕ^	75.6 ± 4.6 ^ϕ^ ***	72.5 ± 6.8 ^ϕ^ ***	46.6 ± 3.1	35.04 ± 2.3 ^ϕ^	73.4 ± 5.1 ^ϕ^ ** ^## $^	62.03 ± 1.3 ^ϕ^ ** ^## $^
SNP								
pEC_50_	7.81 ± 0.09	7.7 ± 0.1	8.07 ± 0.1	8.38 ± 0.07 ^ϕϕ^ ***	8.01 ± 0.06	8.04 ± 0.1	8.7 ± 0.07 ^ϕϕϕ^ *** ^### $$$^	8.54 ± 0.09 ^ϕϕϕ^ *** ^## $$^
Maximal relaxation (%)	94.6 ± 3.09	99.9 ± 1.92	99.9 ± 3.1	94.4 ± 2.18	99.3 ± 3.2	102.1 ± 1.92	96.04 ± 2.4	94.7 ± 1.84
ET1								
pEC_50_	7.02 ± 0.04	6.96 ± 0.04	6.78 ± 0.07	6.85 ± 0.05	6.98 ± 0.04	6.96 ± 0.04	6.84 ± 0.05	6.96 ± 0.08
Maximal contraction (g)	4.57 ± 0.23	4.82 ± 0.3	4.52 ± 0.2	3.34 ± 0.19 ^ϕϕ^ ***	4.57 ± 0.23	4.82 ± 0.3	4.89 ± 0.25	3.44 ± 0.17 ^ϕϕ # $$^

The data are presented as mean ± SE (n = 12 rats). ^ϕ^ *p* < 0.05, ^ϕϕ^ *p* < 0.01, ^ϕϕϕ^ *p* < 0.001 vs. GK−PVAT rats; ** *p* < 0.01, *** *p* < 0.001 vs. GK+PVAT group; ^#^ *p* < 0.05, ^##^ *p* < 0.01, ^###^ *p* < 0.001 vs. GKHFD−PVAT group; ^$^ *p* < 0.05, ^$$^ *p* < 0.01, ^$$$^ *p* < 0.001 vs. GKHFD+PVAT group.

## Data Availability

Data are contained within the article.

## Supplementary Materials

The following supporting information can be downloaded at: https://www.mdpi.com/article/10.3390/ijms25158328/s1.

## Abbreviations

ACh: acetylcholine; AUC, area under the curve; CCL2, chemokine (C–C motif) ligand 2; CCL5, chemokine (C–C motif) ligand 5; CCR2, C–C chemokine receptor type 2; eNOS, endothelial nitric oxide synthase; ET1, endothelin–1; GK, Goto-Kakizaki; HDL, high-density lipoprotein; IPGTT, intraperitoneal glucose tolerance test; ITT, Insulin tolerance test; L-NAME, N-nitro-L-arginine-methyl ester; NO, nitric oxide; PG, propagermanium; PVAT, Perivascular adipose tissue; SNP, sodium nitroprusside; T2D, Type-2 diabetes.
