# Supplementary material for: Propagermanium as a Novel Therapeutic Approach for the Treatment of Endothelial Dysfunction in Type 2 Diabetes
Source: Int J Mol Sci. 2024 Jul 30;25(15):8328. doi: 10.3390/ijms25158328 (PMC11312737; doi:10.3390/ijms25158328)
Supplement: Supplementary file 1 [file ijms-25-08328-s001.zip › ijms-3065904-supplementary.pdf]

Propagermanium as a Novel Therapeutic Approach for the Treatment of Endothelial Dysfunction in Type 2 Diabetes

Lara Azul, Adriana Leandro, Raquel Seiça and Cristina M. Sena *

Institute of Physiology, iCBR, Faculty of Medicine, University of Coimbra, Subunit 1, Polo 3, Azinhaga de Santa Comba, Celas, 3000-548 Coimbra, Portugal; lara.r.s.azul@gmail.com (L.A.); adrianaleandro94@hotmail.com (A.L.); rmfseica@gmail.com (R.S.)
* Correspondence: csena@ci.uc.pt; Tel.: +351-239-480013; Fax: +351-239-480034

Table S1. Blood urea nitrogen (BUN), uric acid (UA), alanine aminotransferase (ALT), aspartate aminotransferase (AST) and alkaline phosphatase (ALP) levels in 8 months-old diabetic Goto-Kakizaki (GK) control rats, GK rats fed with high-fat diet (GKHFD) and GK rats treated with propagermanium (GKPG, GKHFDPG).

	GK	GKPG	GKHFD	GKHFDPG
BUN (mg/dl)	18.5 ± 1.2	14.2 ± 0.5$^{\phi\phi}$	15.8 ± 1.1	12.4 ± 0.2
UA (mg/dl)	1.8 ± 0.3	1.6 ± 0.2	1.7 ± 0.3	1.4 ± 0.1
ALT (U/L)	62.0 ± 5	41.2 ± 5.4$^{\phi\phi}$	68.1 ± 3.4	43.4 ± 2.8$^{\#\#}$
AST (U/L)	164.3± 23	127.5± 8.4	181.7 ± 11.3	142.6 ± 11.1
ALP (U/L)	209.3 ±7.3	158.2 ± 6.1$^{\phi}$	437.0 ± 37.1$^{\phi\phi\phi}$	217.0 ± 18.6$^{\#\#\#}$

Data are expressed as mean ± SE (n = 12 animals in each group). ϕP<0.05, ϕϕ P<0.01, ϕϕϕ P<0.001, vs GK rats; ##P<0.01, ###P<0.001 vs GKHFD.

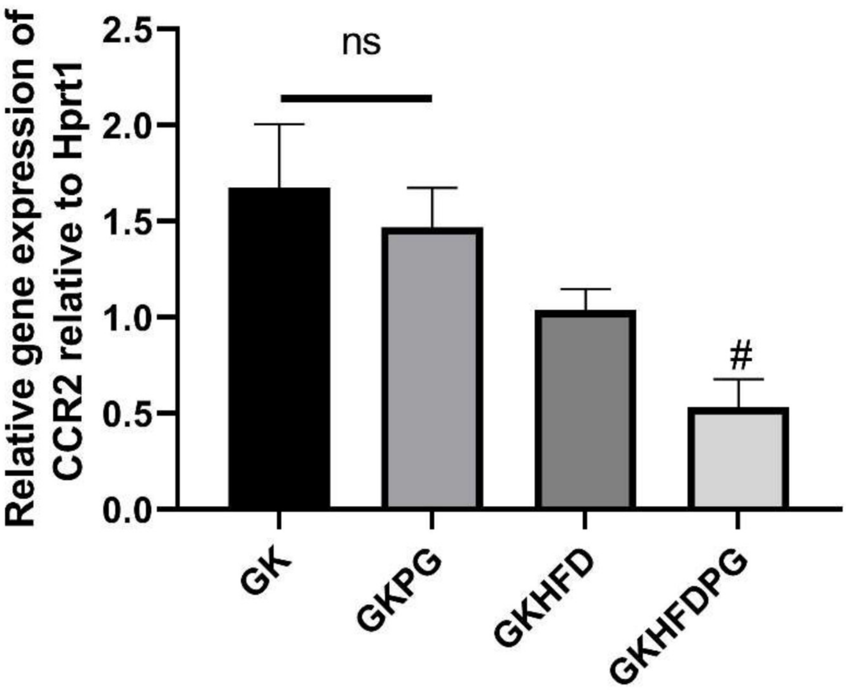

Figure S1. mRNA expression levels of CCR2 gene in PVAT of thoracic aortas of 8 months-old diabetic Goto-Kakizaki (GK) control rats, GK rats fed with high-fat diet (GKHFD) and GK rats treated with propagermanium (GKPG, GKHFDPG). The mRNA expression was studied with quantitative polymerase chain reaction as previously [6]. Data are mean ± SE (n = 12 animals per group). #P < 0.05 vs GKHFD group.
